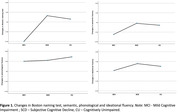# Moderators of language change in middle‐aged and older adults. Preliminary results of the Compostela Aging Study

**DOI:** 10.1002/alz.092312

**Published:** 2025-01-03

**Authors:** David Facal, Cristina Lojo‐Seoane, Alba Felpete, Maria Campos‐Magdaleno, Fátima Fernández‐Feijoo, Benxamin Varela‐López, Yaakov Stern, Arturo X. Pereiro Rozas

**Affiliations:** ^1^ Deparment of Developmental and Educational Psychology, University of Santiago de Compostela, Santiago de Compostela Spain; ^2^ Applied Cognitive Neuroscience and Psychogerontology group, Health Research Institute of Santiago de Compostela (IDIS), Santiago de Compostela Spain; ^3^ Department of Developmental and Educational Psychology, University of Santiago de Compostela, Santiago de Compostela Spain; ^4^ Department of Clinical Psychology and Psychobiology, Universidade de Santiago de Compostela, Santiago de Compostela Spain; ^5^ Cognitive Neuroscience Division, Columbia University, New York, NY USA

## Abstract

**Background:**

Although there is evidence that measures of verbal fluency, naming and word memory can be good predictors of progression to dementia, language change and the main variables predicting it are not yet fully characterized. Recent research draws attention to the need to consider cognitive reserve, functional, and neurobiological indicators together to explain changes (Facal et al., 2021).

**Method:**

The study sample was drawn from the participants of the Compostela Aging Study who completed the third and fourth follow‐up assessments, with about 18 months between assessments. Sample included 24 persons with MCI, 42 with subjective cognitive decline and 41 cognitively unimpaired participants. Language performance was assessed via Boston naming test, semantic, phonemic, and ideational fluency. The difference in performance between the assessments (T4‐T3) was calculated. General linear regression (GLR) models were developed, including the change in each language variable as dependent, and years of education, gait speed (timed‐up and go test), grip strength and mean cortical thickness in the left and right hemispheres as covariates.

**Results:**

Though cross‐sectional differences were found in the four language measures, no differences were found in language change measures (Figure 1). The GLR model with the Boston naming test as the dependent variable was the only significant (F = 5.52, *p*< .01, η2 = 0.26, B‐1 = 0.99). Of the five covariates included in the model, grip strength was significant (F = 21.17, *p*< .01, η2 = 0,21, B‐1 = 0.99).

**Conclusion:**

Naming tests can be good markers of linguistic change in the continuum from healthy cognitive aging to dementia (Flores‐Vázquez et al., 2022). Although this is a first approximation, and 18 months can be a too short time to register significant changes, this work stress the importance of jointly analyzing cognitive reserve, functional and neurobiological predictors.

**References**:

Facal, D., Burgo, C., Spuch, C., Gaspar, P. & Campos‐Magdaleno, M. (2021) Cognitive Frailty: An Update. *Frontiers in Psychology 12*,813398. https://doi.org/10.3389/fpsyg.2021.813398

Flores‐Vázquez, J.F., Contreras‐López, J.J., Stegement, R., Castellanos‐Maya, O., Ćurčic‐Blake, B., Andrés, P., Sosa‐Ortiz, A.L., Aleman, A., Enriquez‐Geppert, E. (2022). Extended FNAME performance is preserved in subjective cognitive decline but highly affected in amnestic mild cognitive impairment. *Neuropyshology 37*, 6, 650‐660. https://doi.org/10.1037/neu0000874